# “If it wasn’t for them, I don’t think I would be here”: experiences of the first year of a safer supply program during the dual public health emergencies of COVID-19 and the drug toxicity crisis

**DOI:** 10.1186/s12954-024-01029-3

**Published:** 2024-06-07

**Authors:** Gillian Kolla, Bernie Pauly, Fred Cameron, Heather Hobbs, Corey Ranger, Jane McCall, Jerry Majalahti, Kim Toombs, Jack LeMaistre, Marion Selfridge, Karen Urbanoski

**Affiliations:** 1 Memorial University, St. John’s, Canada; 2 Canadian Institute for Substance Use Research, University of Victoria, Victoria, Canada; 3 SOLID Outreach, Victoria, Canada; 4 AVI Health and Community Services, Victoria, Canada

**Keywords:** Prescribed safer supply, Safer supply programs, Overdose, Fentanyl, Opioids, Stimulants

## Abstract

**Background:**

In response to the devastating drug toxicity crisis in Canada driven by an unregulated opioid supply predominantly composed of fentanyl and analogues, safer supply programs have been introduced. These programs provide people using street-acquired opioids with prescribed, pharmaceutical opioids. We use six core components of safer supply programs identified by people who use drugs to explore participant perspectives on the first year of operations of a safer supply program in Victoria, BC, during the dual public health emergencies of COVID-19 and the drug toxicity crisis to examine whether the program met drug-user defined elements of an effective safer supply model.

**Methods:**

This study used a community-based participatory research approach to ensure that the research was reflective of community concerns and priorities, rather than being extractive. We interviewed 16 safer supply program participants between December 2020 and June 2021. Analysis was structured using the six core components of effective safer supply from the perspective of people who use drugs, generated through a prior study.

**Results:**

Ensuring access to the ‘right dose and right drugs’ of medications was crucial, with many participants reporting success with the available pharmaceutical options. However, others highlighted issues with the strength of the available medications and the lack of options for smokeable medications. Accessing the safer supply program allowed participants to reduce their use of drugs from unregulated markets and manage withdrawal, pain and cravings. On components related to program operations, participants reported receiving compassionate care, and that accessing the safer supply program was a non-stigmatizing experience. They also reported receiving support to find housing, access food, obtain ID, and other needs. However, participants worried about long term program sustainability.

**Conclusions:**

Participants in the safer supply program overwhelmingly appreciated it and felt it was lifesaving, and unlike other healthcare or treatment services they had previously accessed. Participants raised concerns that unless a wider variety of medications and ability to consume them by multiple routes of administration became available, safer supply programs would remain unable to completely replace substances from unregulated markets.

## Background

Canada remains in a drug toxicity crisis driven by an unregulated opioid supply composed predominantly fentanyl, fentanyl analogues, and more recently, benzodiazepines. The province of British Columbia (BC) has been at the epicenter of this crisis; since federal tracking began in 2016, BC has consistently had the highest rate of death among Canadian provinces and territories, with a death rate of 46.2 per 100,000 population and with fentanyl detected in 85% of the 2,551 drug toxicity deaths in 2023 [1]. The province first declared a public health emergency due to rising levels of overdose deaths in April 2016, with 13,000 drug toxicity overdose deaths since then [2]. The rate of drug toxicity deaths across Canada escalated with the start of the COVID-19 pandemic in 2020; while a small number of safer supply programs existed prior to the pandemic, additional safer supply programs were scaled-up during this period to attempt to address rising rates of drug toxicity deaths [3–5].

In Canada, the terms ‘safe supply’ and ‘safer supply’ have been used interchangeably to describe a variety of program models and approaches to providing substances (primarily pharmaceutical opioids, stimulants and benzodiazepines) of known dose and composition to people who would otherwise be solely reliant on procuring substances from the unregulated market, ranging from medicalized, prescriber based models to grassroots efforts for non-medicalized models (e.g., compassion or buyer’s clubs) [3, 6, 7]. There have been attempts to obtain state-sanction for non-medicalized compassion club models that distribute substances of known composition to members [8–10]. Despite evidence that participation is associated with fewer non-fatal overdoses [10, 11], the federal government has not approved exemptions from federal legislation for compassion clubs to operate as sanctioned non-medicalized models. As such, the scale-up of sanctioned safer supply models has been limited to medicalized models where authorized medical professionals (physicians and nurse practitioners) prescribe pharmaceutical medications through existing regulatory regimes to people who are using and reliant on the unregulated drug supply [5, 12]. Prescribed safer supply had been occurring on a small-scale prior to the COVID-19 pandemic, with small programs in Ontario since 2016 [4, 13]. However, there was a rapid increase in safer supply prescribing during the COVID-19 pandemic, with a 285% increase in programs documented between March and May 2020 [3]. This corresponds to the onset of the COVID-19 pandemic, where Risk Mitigation Guidance (RMG) was released in March 2020 in BC as a form of safer supply that supported the prescription of take-home doses of opioids, benzodiazepines and stimulants to people reliant on the unregulated drug supply to facilitate COVID-related isolation measures and reduce the risk of fatal overdose during pandemic-related isolation periods [14].

Concomitant to this increase in safer supply prescribing, there has been a rapid increase in the evidence base on the impacts of safer supply prescribing, with research and program evaluations reporting: reductions in unregulated drug use and overdose risk among program participants; client-reported improvements to physical and mental health functioning; improvements in social outcomes such as financial and housing stability; and an increased sense of personal stability and control over their drug use [13, 15–21]. These self-reported outcomes are aligned with analyses of health administrative data comparing safer supply program participants and matched individuals with opioid use disorder not receiving safer supply; significant reductions in clinical measures (emergency department visits, hospital admissions, admissions for infectious complications and health care costs) were found in a study from the province of Ontario [4]. In a separate study of BC administrative data, there were significant reductions in overdose mortality (55–89%) and all-cause mortality in BC (61–91%) in the week following receipt of safer supply medications, compared to matched controls [22].

Previous research has highlighted the need to center the perspectives of people who use drugs in safer supply program development and delivery, documenting six core program elements that drug users consider to be core components in an effective safer supply model: (1) Right dose and right drugs for me; (2) Safe, positive and welcoming spaces; (3) Safer supply and other services are accessible to me*;* (4) I am treated with respect; (5) I can easily get my safer supply; (6) Helps me function and improves my quality of life (as defined by me) [23]. In the present study, we use these core components to explore participant perspectives on the first year of operations of a safer supply program in Victoria, BC, to examine whether the program met drug-user defined elements for an effective safer supply model.

### Program description

The Victoria Safer Alternatives for Emergency Response (SAFER) initiative is a community-based safer supply program that is operated by AVI Health and Community Services in collaboration with SOLID Outreach, a local organization of people who use drugs. While SAFER began early in the COVID-19 pandemic in June 2020, the program model had been developed and funding applied for in 2019 from Health Canada’s Substance Use and Addiction Program [20]. The SAFER model combines prescriptions for safer supply of prescription opioids and stimulants with comprehensive harm reduction programming, the provision of primary care and social services, and access to addiction medicine where desired in an integrated program as part of an effort to reduce the risk of overdose. During the implementation of the SAFER program, the limitations of the RMG were quickly realized by SAFER clinical and outreach staff, who wrote their own protocols and practice brief based on their experience and feedback they were receiving from participants in the program [20].

The SAFER program initially targeted populations of people who were using drugs and experiencing homelessness, primarily those living in informal encampments in parks following eviction from shelters during the early pandemic period. Driven by COVID-related service closure and restrictions on in-person service delivery, the initial SAFER model involved outreach by staff (primarily a registered nurse and outreach workers from SOLID outreach with lived or living experience of drug use) into encampments to ensure that people using unregulated drugs at high risk of overdose and who were unhoused would have access to safer supply prescriptions. Eligible people were connected with SAFER prescribers and provided with prescriptions of short-acting pharmaceutical opioids (oxycodone and hydromorphone, often referred to by the brand name Dilaudid or *dillies*), as well as long-acting formulations such as slow-release oral morphine (often referred to by the brand name Kadian) and fentanyl patches, as well as prescription stimulants depending on their needs. Medications were provided alongside low barrier health services (frequently directly in encampments) to meet a wide variety of health needs people. System navigators also worked with participants to access housing and income supports. In June 2021, the SAFER program acquired a storefront, allowing for a fixed site for the provision of clinical and social services.

## Methods

This study used a community-based participatory research (CBPR) approach to generating and analyzing data that is actionable. Continuing with the approach used in the concept mapping study mentioned above [23], people who use drugs (including SAFER program participants and members of the local drug user organization SOLID) were involved in all aspects of the research process, from conception and design, to data collection, analysis, and reporting [24, 25].This was done to ensure that the research was reflective of community concerns and priorities, rather than being extractive [26, 27]. It also ensured that the evaluation reflected the perspectives of program participants and not only service providers and decision-makers.

In order to centre service user experiences and perspectives in the design and delivery of the program, a SAFER research and evaluation team composed of people with lived and living experience of drug use partnered with SOLID outreach (a local drug user organization), AVI (the organization running the SAFER program) and academic researchers from the University of Victoria to use concept mapping to develop a model for safer supply service delivery developed by people who use drugs [23]. This study uses these six clusters to evaluate the implementation of the SAFER program from the perspective of program participants. Exploration of participants’ experiences in SAFER is critical to understanding factors that both facilitate and act as barriers to program involvement and successful outcomes as defined by service users.

We used qualitative methods to gain insight into the participants’ experiences with and opinions of the SAFER program. This study formed part of a larger provincial evaluation of the implementation and impacts of the risk mitigation prescribing in BC [28]. As part of the provincial evaluation, 55 people who use drugs and who had received or were trying to access a risk mitigation prescription were interviewed about their experiences and impacts. SAFER participants were over-sampled to permit a dedicated program evaluation. In total, 16 participants identified as SAFER program participants, generating the sample for this study.

Participants were interviewed between December 2020 and June 2021. Interviews were conducted in-person and by telephone. In-person interviews were conducted in homeless encampments. Participants received an honorarium of $30 CAD. Interviews were audio recorded, open ended and lasted between 45 and 60 min. Transcripts were imported into NVivo (Version 12) to facilitate coding. Data were coded by several members of the writing team who met regularly to review transcripts and develop initial codes in the data and inductively develop a coding framework. The analysis was structured according to the six core components of effective safer supply from the perspective of people who use drugs, generated through a prior study conducted to inform the development of the SAFER program [23]. Ethics approval for this study was obtained from University of Victoria (#20–0293).

## Results

### Participant description

All participants interviewed for this study were receiving prescribed safer supply through the SAFER program. Of the 16 participants, 14 self-identified as men and two self-identified as women. Six participants identified as Indigenous and 10 did not disclose their race/ethnicity. Out of the 16 participants interviewed, only two reported having stable housing; the remaining 14 reported unstable housing consisting of living in homeless encampments, COVID-19 sheltering sites (frequently re-purposed hotels), shelters and couch surfing. Three participants reported having completed some college education, six had completed high school and four had not completed high school. In terms of drugs used, eight participants reported using opioids only, three reporting used stimulants only and five reporting using both opioids and stimulants. None reported using benzodiazepines knowingly.

#### Component 1: right dose and right drugs for me

This first component of effective safer supply focuses on “the importance and availability of the right drugs in the right dose via the right route” (p. 3, 21) to enable program participants to decrease or cease their use of substances from unregulated markets.

### Availability of multiple medication options

There was substantial variation in the medications and/or medication combinations that SAFER participants found effective. As one person receiving short-acting oxycodone remarked: *“It helped me through my dope sickness and stuff like that. And withdrawal. But yeah, like I said, the milligrams are just right, and how much they give me is perfect so far”* (2970).

Another person receiving oxycodone also found their dose to be effective, but experienced varying results due to the different modes of administration. Specifically, they noted a difference between oral use of medications versus smoking or injecting unregulated opioids, which produced a more rapid onset of effects: “*The strength seems pretty much good. Be nicer to have a little bit more quick release. It sort of takes a while for them to kick in*” (2005).

One of the advantages of safer supply was that participants were able to have input regarding their dose, with flexibility to help find the right combination of medications that would work for them. One participant receiving 12 tablets of 8 mg short-acting hydromorphone daily reported that they were “very satisfied” with their dose, saying: “*I believe they can go much higher. I just didn’t want to go much higher myself. The least amount possible in my eyes is better. Then it’s the less damage on my body*” (2260). Other participants reported needing a combination of both short and long-acting opioids, suggesting that a combination was necessary to address their withdrawal, as reported by this individual receiving short-acting oxycodone tablets and slow-release oral morphine: *“I just find it’s better for me. And more of a stronger, right? Helps my withdrawal. I was using a lot and now it’s kind of like gotten a lot better*” (2305).

SAFER began offering fentanyl patches as an option in their first year of operation, and several participants reported finding them effective to manage pain and withdrawal (dope sickness): “*the pills are helping with my pain that I have regularly, but they don’t help any way with the dope sickness, which is why I got put on the fentanyl patch, which is starting to help tremendously”* (3475). The variety of different medications available to participants seems crucial in meeting a wide variety of individual needs, while also decreasing reliance on unregulated substances, experiences of withdrawal, and pain.

Participants identified a need for a safer supply of stimulants. A smaller number of participants reported receiving prescribed stimulants, with varying degrees of success. One participant reported success in stopping using crystal methamphetamine after receiving stimulant safer supply, to a degree that surprised even them: “*It’s a big change for me so far. I’m still baffled on how I actually kicked the crystal. I didn’t think it would be that easy*” (2970). Others reported decreasing their use of unregulated stimulants: “*And same with Adderall. Like it’s cut my use in half of meth*” (2896). These findings highlight that when the right drugs are provided in the right dose, program participants experienced positive impacts of prescribed medications.

### Name brand versus generic hydromorphone

One of the major issues identified with the available formulations of hydromorphone related to differences between the generic and brand name (i.e., Dilaudid) tablets. Unanimously, participants did not feel that the generic formulation worked as well as the brand name, with one participant stating, “*generic sucks*” (2005). Some felt that the quality of the generic hydromorphone was inferior: “*The quality’s not that great with generic*” (2305), while others questioned the effectiveness of generic brands: “*the generics didn’t work for me,”* (2260) and: “*We need the brand name ones. They’re a lot better. I find they work a lot better*” (2305).

### Issues obtaining appropriate strength and substitution from medications

While many participants reported success with the available pharmaceutical options, others highlighted issues with the strength of the available medications. Participants raised concerns that without a wider variety of medications and multiple routes of administration, safer supply programs would remain unable to completely replace substances from unregulated markets. Some SAFER participants did not find hydromorphone effective, with one participant stating: “*Well, dillies [slang term for hydromorphone], I just found I didn’t feel them at all…it just wasn’t really doing anything for me*” (2896). Another participant highlighted that for those who had extensive exposure to fentanyl from the unregulated market, substituting opioids instead of directly providing fentanyl products was not effective: “*The body wants fentanyl, not oxycodone*” (3475). Issues with obtaining a suitable strength of medication was noted as challenging in the context of an increasingly potent supply of unregulated fentanyl and associated increased opioid tolerance among individuals.

Similarly, the strength of available stimulant options was noted by some participants as a problem, with one commenting about dextroamphetamine: “*It’s keeping my body in line, but it’s not stimulating it. No, not like I would off of the street meth*” (3251). Another participant concurred, saying of currently available oral stimulants: *“It lacks that power. It lacks the real kick. Not the same as side [crystal meth*]” (3287). Another participant rightly noted the lack of ‘right drug’ options available for prescribed stimulants, namely, the lack of pharmaceutical formulations of cocaine, methamphetamine or amphetamines:

But it [safer supply] suits the opioid community more than it does any other community. Because there is no—there’s nothing for speed. Or sorry, there’s nothing for cocaine. Like at least the [hydromorphone] is, like an actual opiate, right? Whereas the speed is just like, it’s not real speed, right? It’s the closest thing to. So it’s kind of like, it’s a substitution? So I guess just maybe trying to get the real thing (3287).

This participant calls for the program to “get the real thing” for prescription options, which can more directly replace substances people are able to access through unregulated markets.

### Lack of options for people wishing to smoke their medications

Finally, while some people who had previously smoked fentanyl were content with a switch to oral use of tablets, others had tried to crush and inhale/smoke the hydromorphone and found it impossible to get a high from this mode of administration. When asked about their experience trying to smoke hydromorphone, one participant stated “*there’s nothing at all*” (2260). Another participant specified that lack of options for different modes of administration was a barrier to safer supply, and might result in diversion: “*They can’t snort it, they can’t smoke it. So it’s just, it’s useless. They won’t take it right?”* (2387). Inability to smoke medications was also an issue for the available stimulant options. One participant reported success in decreasing their use of crystal methamphetamine after being prescribed dextroamphetamine (i.e., Dexedrine), however they found the mode of use problematic: “*I already tried smoking it and it just didn’t work*” (2970). Participants repeatedly noted the lack of smokeable medication options for people who smoked opioids or stimulants as a major shortfall of safer supply.

#### Component 2: safe, positive and welcoming spaces

The second component of effective safer supply reflects the importance that participants placed on receiving care in spaces that were non-stigmatizing, and where they felt that they were safe, welcomed and valued.

### Non-stigmatizing access to care

Overwhelmingly, participants reported receiving compassionate care, and that accessing the SAFER program was a non-stigmatizing experience. Participants highlighted the kind treatment they received from staff members as a key factor in making them feel welcome: “*I love it, they’re great. You know, such nice people. I don’t know where you guys find these people, but they got such good hearts”* (2005).

This contrasted with previous negative experiences within the healthcare system, where participants felt that they were not treated with respect due to their drug use: “*They’re nice, they’re actually trying to help us and they don’t treat us like addicts, you know?”* (2305). One participant described their experience trying to access safer supply at an opioid agonist treatment clinic prior to becoming a SAFER participant: “*They’re just really rude, and they’re just like ‘Oh yeah, you’re just trying to get high off it’*” (2305). This illustrates the challenges of trying to access safer supply, even in the one province with interim clinical guidance for providing safer supply prescriptions during the COVID-19 pandemic (RMG), as well as how people experience stigma, even in addiction treatment settings.

### Participant feedback central to program

Participants felt strongly that the feedback of people who use drugs should be central to the design and implementation of safer supply programs as part of the ongoing response to the drug toxicity crisis. One individual noted the urgency of including the expertise of people who use drugs who need to “…*get a voice. And if we get a voice, maybe we’ll get some change. And if we get some change, maybe people will stop dying*” (3794). Participants also felt that the SAFER program had been effective in soliciting feedback from participants: “*The SAFER people that come and see us, they always ask us if we need to improve anything*” (2305). Participant feedback was used for quality improvement to ensure that the program remained safe and welcoming for participants, and to give participants space to highlight what program elements were working well.

### Need for long-term program sustainability

Participants were frequently aware of the contentious nature of safer supply programs, and several expressed fear about what they would do if the program was cancelled: “*You know, people bitch and shit about having it right now, but without it it’s going to be a hell of a lot worse*” (2005). In addition to fears about whether the program would continue, when asked about what they would do if safer supply programs ended, participants highlighted the lack of other options available to them, other than returning to procuring fentanyl from the street supply: *“I guess I’d be stuck to the fentanyl. It’s…That’s it. Completely depressed. And, stuck to the thing that I want to get rid of” (2260).* This concern continues since safer supply programs are funded on a short-term, pilot basis, with federal funding currently extending only to March 2025 for existing programs.

#### Component 3: safe supply and other services are accessible to me

The third component of effective safer supply programs underscores accessibility which was demonstrated through outreach, coordination of care and various health and social supports being accessed through one program.

### Outreach facilitated program involvement

Most participants indicated that they learned about SAFER through an outreach worker or a nurse. They were generally approached while they were living in encampments, or at their place of residence (COVID-hotel, shelter or at their tent in a city park). One participant described the experience of meeting the SAFER team member:

I was living on the street and everything else. I didn’t know anybody, so I had no way of getting anything. You know, they were walking through the parks and they come up and they’re offering to help. The street nurse and then the safe supply, the needles, and the cookers, and everything else. And they’re helping me get prescriptions, helping me get to the doctors and all that. They’re just - to me they were just something that was unbelievable. It’s great for people who are like living on the street and that don’t have nothing left. (3676)

At the beginning of the SAFER program, outreach teams consisting of nurses, harm reduction workers, and people with lived experience would take medications out to some participants and dispense them in community, particularly in the encampments in the early pandemic period.

And like they come to wherever you are usually, right? So, it makes it easier if you’re homeless. You don’t have to take a chunk out of your day, just figuring out how that’s all going to work, right? Going to wherever the doctor is. Just having to deal with that. And a lot of the time you don’t have the resources, like you don’t have a car, so you got to ride the bus there, you might not have change for it. Or a phone to figure out the directions, you know. It can be difficult. (2286)

For many participants, this was a very successful strategy to connect them to care, particularly for people who were disconnected from the healthcare system and community services. By visiting participants in the community, outreach workers removed the burden of transportation and navigation.

#### Coordination of care

Participants also appreciated the ability to be connected to a variety of services through a single provider. Instead of navigating several agencies on their own, SAFER staff communicated amongst themselves and assisted participants to access appropriate care as needed:

Just there’s so many more different teams trying to help out, and I think they communicate quite a bit. And how they get in touch with the pharmacies and doctors, no problem. (2005)

Personal connections between providers and participants further facilitated access to care, particularly when so many agencies and services were closed due to COVID-related public health restrictions. Participants described feeling the sense of teamwork and trust among SAFER staff. For instance, one participant described how nurses facilitated access to prescriptions for them:

[SAFER Nurse] just gave them my information and the doctor trusted [SAFER nurse] and they just made the prescription. I got it over the phone. I think it’s [the accessibility] great. I think as long as you know someone that you can get in contact with, that can get someone on that thing, you’re in luck. I mean, as long as you have that in. But if you don’t have that in—it could be very hard, very difficult. (3287)

Assisting participants to identify and navigate the diverse support and care options available to them through the program further enabled uptake.

#### Access to wrap-around care and other services

Beyond safer supply prescriptions, participants were appreciative of efforts by outreach workers to find housing, access food, get their ID, and support any other needs. One participant reported that staff helped him with “g*etting all my ID back*” (2260), which can be critical to accessing other services (i.e., provincial government health insurance). When asked about the process of getting housed, another participant explained various ways that service providers intervened and supported the process: *“…they helped me with paperwork, and they were the ones that introduced me to the right people to talk to and help me get going with it*” (3676). Another participant explained that workers helped with *“…getting our birth certificates, for instance. Just little things, like getting a doctor’s appointment, getting six Ensure [meal replacement drink] a day*.” (2896).

#### Difficulties in initial access and limits to program capacity

Though the outreach model described above facilitated access for many, it also created challenges for potential program participants as there was no clear access point or person to get more information from regarding SAFER. Responding to a question about knowing how to access the program, one participant said: “*No, that’s the problem, right? I didn’t know where to go or how to do it, right?”* (2305) This same participant recalled learning about the program when SAFER staff happened to pass by their tent, highlighting an element of chance in accessing the program.

Program capacity limits driven by budget constraints rendered the program unable to meet the high demand for safer supply in the community. Additionally, due to the medicalization of safer supply, obtaining access to medications necessitated entering into a prescriber-patient relationship that many prescribers were either unwilling, or did not have capacity, to take on. Participants who were able to access safer supply expressed that they were grateful to be part of the program and thought the program could help many others who were struggling to find a prescriber. “*More people like me that have problems seeing a doctor. Well because I know a lot of doctors aren’t taking new patients right now*” (2297). Demand for safer supply surpassed the availability of prescribers in the community, even in the height of the COVID-19 pandemic when rates of fatal overdoses peaked. The initial scale-up of the SAFER program as a pilot program during the height of the COVID-19 pandemic meant that accessing the program was often not an easy process, as there was a lack of coordination and direction from the broader healthcare system given challenges during that time period.

#### Component 4: I am treated with respect

The fourth component of safer supply effectiveness focuses on respectful treatment of participants as shown through good communication and building trusting relationships.

#### Providers treated me with respect

Participants talked about the care they received from SAFER staff, and appreciated how providers would find them if they missed their medications. One person reflected on how they had struggled to navigate services on their own prior to being part of SAFER, and now: “*it’s nice to have the help. And have someone there that it actually feels like somebody cares to help”* (2260). Another participated remarked: “*I’ve never had anybody like them, or AVI and SOLID, the SAFER team that comes and sees me now. I never, ever thought I’d have people like that*” (2970).

SAFER participants felt respected in interactions with their service providers and described them as going above and beyond: “*I was dealing with Nurse [name of SAFER nurse]. They were really, really, fast with me and good. And they made the prescriptions come right away. Like they were above and beyond for sure.”* (3287). Overwhelming, participants spoke in glowing terms about the nurses, outreach workers and peer workers, describing them as helpful, non-judgmental, respectful, and generally positive compared to other healthcare.

#### Developing relationships and trust

Participants highlighted how SAFER staff made a point of building relationships and personally connecting with participants, which further facilitated respectful, trusting care:

They [SAFER staff] just goes out of their way on just anything. Helped me with -so far- with my ID, my PWD [disability income support], just making sure I keep up, on par with my meds. And they’re a good listener. I’m still grateful that I actually met them. And if it wasn’t for them, I don’t think I would be here. (2970)

Beyond individual interactions, participants also described feeling looked out for on a regular, ongoing basis by SAFER staff: *“I feel like I’m being watched, looked out for, which makes me feel good*.” (3475) Service providers went out of their way to stay in touch with the SAFER participants: “*The people that go out and walk through the tent cities and things like that. You know, they’re out there trying to find the people and help them and things like that. That’s where it helps.”* (3676).

Having trusting relationships with care providers was a new experience for many participants, who had previously felt judged and surveilled when accessing services. One participant distinguished their experience at SAFER with their experience of receiving methadone:

I like the trust they have in us to take them [medications]. Which is fair. I’m taking—I want to take them to get off of the initial drug anyways. There shouldn’t be the need to have somebody watch me take them. Whereas methadone, they would actually have to watch me take the methadone. I’m not allowed to take that home. (2260)

Beyond specific services, outreach workers also knew and facilitated personal supports for participants relative to their specific needs, such as reconnecting with family members:

…they [SAFER staff] called my sisters for me. Yeah, I was too scared to call my sisters and tell them I loved them because I had no good news for them, right? And I know they love me and all they want to hear is my voice, and tell them I love them. But I just couldn’t do it this time. And we’re pretty close, so they did it for me. (2005)

The variety of personalized supports provided by workers—from paperwork to housing to family connections– illustrate the importance of respectful participant-provider relationships which further facilitates the provision of safer supply.

#### Component 5: I can easily get my safer supply

The fifth cluster focuses on factors for obtaining safer supply on a regular and continuous basis, specifically where medications were picked up and how often, as well as experiences with witnessed versus take-home doses.

#### Daily pick-up versus delivery of medications

Participants typically picked up their medications daily at a pharmacy. Multiple participants expressed difficulties making it to the pharmacy for multiple reasons; some were experiencing extreme accessibility barriers due to mobility challenges, chronic health conditions and mental health challenges, while others had a hard time with daily pharmacy visits because they were sheltering in encampments and were at risk for having their survival supplies (e.g. tents, sleeping bags) confiscated by bylaw officers and police. Due to these factors, participants spoke of the burden of integrating pharmacy pick-ups into their schedules: “*Because I missed a few of my days just because it’s not been convenient to the rest of what I’m doing, to just go pick it up*.” (2286) Some pharmacies offered daily delivery of medications, including observation at time of delivery of daily doses of long-action medications like methadone and slow-release oral morphine. This was expanded during the COVID-pandemic period to facilitate isolation, but also helped to increase accessibility for people with mobility impairments where daily trips to the pharmacy constitute a barrier to access. Participants could choose to have medication prescriptions sent to their pharmacy of choice, and as such could choose a pharmacy based on location or whether the pharmacy had delivery available. Additionally, in the first year of the SAFER program, outreach teams would also deliver medications to participants, particularly those living in encampments. There were pros and cons to daily trips to the pharmacy vs. delivery. Many participants found daily pharmacy trips to be disruptive: “*I go to pick it up. I want to have a delivery thing, but it’s daily. Sometimes it’s a real hassle to go pick it up. And then it spins you out because if you don’t go pick it up, then 24 h later, you’re really regretting it.*” (2471) This quote also highlights how pharmacy delivery could also be problematic, as missing the medication drop-offs meant that participants could not get their dose that day.

However, some participants appreciated the delivery of medications: “*Ah, it was hard for us to go to the pharmacy and pick it up. So they had to start delivering*” (2297). In particular, delivery may help some participants overcome accessibility barriers due to experiencing homelessness. Conversely, in a few cases, participants appreciated having to go to the pharmacy which provided some routine: “*The structure is probably a good thing. I like the structure, not having them all at the same time and kind of having a schedule. You know, you have to go there every day to get them.”* (2615). Some participants felt that daily pick-ups helped them regulate the amount of medications they had in their possession at once.

Some expressed feeling alienated when picking up their prescriptions from the pharmacy: *“The pharmacy themselves, they’re a little bit snooty. But I mean, what can you do? They give me kind of looks sometimes, like ‘Oh, it’s the safe supply guy.’ You know what I mean?*” (3287) This highlights how even if participants experience respect and acceptance within the SAFER program, they could still be subjected to stigma within the community, potentially impacting their ability to get their medications.

#### Observed versus take-home doses

Many participants were required to have a pharmacist observe them taking some of their medications daily at the pharmacy (particularly long-acting opioids like methadone or slow release oral morphine). The rest of participants’ medications were prescribed as take-home doses that did not require observation; however, daily pick-ups remained onerous:

…it [safer supply prescription] should be weekly. I don’t suggest it for everybody, you know what I mean? It depends on where the people are at. If someone’s in a healthier place because of their commitment to their prescription, then it shouldn’t be so rigidly…yeah, it’s stupid. (2471)

There was a desire for weekly pick-ups to be available, especially for those who had been on the program for a while and had demonstrated stability. The requirement for observed dosing was also closely tied to feelings of autonomy, or lack thereof.

You’re forcing people to use drugs at a set time every day. What happens if they have a family dinner at 4 o’clock and they pick up their meds at 3:30? They’re not going to want to be half out of it. You’re forced to use drugs at a certain time every day. (3794)

Comparatively, take-home doses enabled participants to autonomously manage their needs.

#### Component 6: Helps me function and improves my quality of life

Lastly, the sixth component emphasizes the various and intersecting ways that safer supply programs are improving participants’ overall quality of life as defined by their individual needs and goals.

#### Reduced reliance on the unregulated drug market and reduced overdoses

Several participants highlighted that accessing the SAFER program allowed them to reduce their use of drugs from unregulated markets, with one participant receiving short-acting hydromorphone remarking: “*When I first started the dilly-8s [hydromorphone 8mg tablets], I started smoking fentanyl a lot less*” (2260). Another participant noted that after starting the safer supply program, their fentanyl use decreased substantially, though it did not provide the same effects: “*It cut my use in half*. *But it doesn’t have that same sensation of euphoria or actually feeling high”* (2896). Other participants who had previously been smoking fentanyl appreciated the transition to oral medication, and found that oral opioid medications resulted in both euphoria and reduced the amount of fentanyl they smoked substantially: “*Yeah, it did. It got me high and I don’t have to think about actually smoking my brains out anymore. And it definitely did the job. And I’m still grateful for it today*” (2970).

Accessing safer supply was also effective for avoiding overdose, as participants could “*access what I need*” (2297) without using street-acquired fentanyl of unknown content and strength. Participants reported no or fewer overdoses since beginning safer supply; for example, one participant who had experienced eight overdoses while using street-acquired fentanyl had not experienced an overdose since starting on SAFER: “*Not a single one*” (2260).

#### Managing withdrawal, cravings and pain

For many participants the purpose of safer supply was to treat the symptoms of withdrawal: “*I think it’s a good thing, because it stops the dope sick”* (3251). The ability to prevent withdrawal and cravings was crucial to participants feeling better in daily life: “*And I’m able to get up in the morning because [before safer supply] I’d be like feeling just so dope sick, I wouldn’t be able to get up or do anything*” (2305).

Chronic pain was an ongoing challenge for many participants who reported varying degrees of relief from their prescribed safer supply. Several participants were still topping up with fentanyl from the unregulated supply to control their pain: “*I’m doing the street drug mainly now for painkiller*” (3251). For another, avoiding withdrawal symptoms was important, but their current dose did not provide the full relief they required: “*It kind of just took away the dope sickness. It doesn’t take away all the extra pain. I started the opiate to get the pain relief”* (2260).

#### Avoiding the hustle and reduced involvement in criminal activity

Some participants talked about how safer supply reduced their involvement in criminal activities: *“I’m not having to steal or boost or anything to get my drugs*” (3676). Many spoke of how access to safer supply meant they no longer had to constantly hustle for money. One participant reflected that this change resulted in them having more time doing things they enjoyed: *“Not having to make as much money, so a lot less time is spent bottling [collecting bottles and cans to return for refund] and more time sitting around and talking and enjoying my time spent with friends. Which I enjoy doing*” (2260). Another participant talked about how they feared getting cut off from the program because they would have to go back to “*the same thing, the hustling and the grind*” (2297).

#### Achieving personal goals and finding stability

Participants had a variety of reasons for accessing SAFER and described the stabilization they had achieved as helping them to reach their goals for their drug use, health and personal lives. For some, safer supply moved them closer to stopping drug use altogether: “*I guess maybe they kind of give me a thought about maybe changing it. Quit drugs*” (2615). Another participant said “*I’ll be on it until I’m able to kick the dope*” (3475). While some noted they were motivated to eventually stop using drugs altogether, it is important to note that abstinence was not a requirement for participation in the program.

Other participants described a positive shift in their energy levels and mood due to safer supply. For instance, “*I’ve felt more uppity. More energetic, happy that I don’t have to fight to make money to do the thing that I don’t want to do anymore. A lot less depressed*” (2260). This reflects a positive shift in overall wellbeing after getting involved in the SAFER program.

## Discussion

Using the six components of effective safer supply programs as identified and defined by people who use drugs to evaluate the SAFER program’s first year of operation, we found that the SAFER program was effective at helping participants to avoid withdrawal and cravings, reducing use of substances from unregulated markets, and reducing the occurrence of overdose. The program connected with a group of people experiencing homelessness who were largely disconnected from services with respectful, non-stigmatizing and accessible health and social care. While participants identified the need for more options for medications—including access to the actual substances available within the unregulated drug supply for direct substitution—and for formulations of medications that could be administered in different ways (particularly smokeable options), many participants also reported satisfaction with the medications being prescribed. Our findings align with existing research that the expansion of safer supply programs improved accessibility of health care services for a group of people and reduced overdose risk for a group of people at high risk of overdose in the early COVID-pandemic period [3, 13, 19, 21, 22, 29, 30].

The extensive outreach employed by SAFER staff directly in encampments to people deprived of housing and sheltering options was key to making safer supply accessible. Our results highlight how providing medical and social services in encampments allowed SAFER staff to connect with people who would have otherwise not been able to access safer supply prescribing, or any medical care or social services in the context of COVID-19 related restrictions. BC Coroner’s Service data have reported found a strong association between drug toxicity overdose deaths and experiencing homelessness and/or housing instability [31] and data from Ontario show a 139% increase in drug toxicity deaths among people experiencing homelessness during the first year of the COVID-19 pandemic [32]. Additionally, our results suggest that outreach was crucial in averting overdose deaths among a highly vulnerable population not being served by traditional health care or addiction treatment models.

Similar to other studies exploring safer supply program scale-up in Canada [3, 5], our study also identified key barriers to accessibility, particularly the need for observed dosing of long-acting opioids and daily dispensing of short-acting opioid medications. These challenges also exists in opioid agonist treatment (OAT) programs, and people receiving OAT have repeatedly highlighted the barriers to accessibility created by observed dosing and daily pharmacy visits [33–35]. Evaluations of injectable OAT programs have highlighted how multiple daily visits to consume medications under observation are onerous, reduce program retention and hinder the ability of participants to attend school, obtain employment or travel [36–39]. And while there are a wide variety of OAT service models, conventional OAT practice has tended to promote abstinence from injection drug use and/or from using street-acquired opioids to experience euphoria (particularly for clients wishing to ‘earn’ take-home doses); in contrast, prescribed safer supply has centred harm reduction goals and aims to provide an alternative to the unregulated supply. Additionally, the medications used in OAT programs and the strict requirements around them may not be in line with the goals or preferences of people who use drugs, some of whom do not wish to interact with the addiction treatment system for a variety of reasons, including previous discrimination and negative experiences [7, 40, 41]. The COVID-19 pandemic allowed for a form of natural experiment, as in some jurisdictions both OAT and heroin-assisted treatment (HAT) programs allowed for more take-home doses and loosening of requirements that all opioid medications be taken under observation [42–46]. This resulted in stronger retention to treatment seen among OAT clients who received more take-home doses, and low rates of adverse events in both OAT and HAT programs [44, 45, 47]. While take-home doses are frequently restricted due to concerns about diversion, these results suggest that the longstanding critiques of strict observed dosing models from people who use drugs have merit and the emerging results from safer supply programs may be usefully applied within OAT programs to reduce barriers to care and allow for more flexibility for people accessing OAT. Additionally, research suggests that co-prescription of safer opioid supply alongside OAT can sustain retention to OAT [48, 49]. Future research should seek to examine the contexts and circumstances where flexibility and increased access to take-home and non-observed dosing may lead to increased autonomy and flexibility for people receiving safer supply, iOAT and OAT. Participants in our study also highlighted that being able to access prescribed medications that are the right drug in the right dose by the right route would reduce diversion, as people are not diverting medications when they are able to receive prescriptions that meet their needs.

Overwhelmingly, participants highlighted that they received respectful, compassionate care from the SAFER program, and that the trusting relationships with care providers was markedly different then their experiences accessing other healthcare or addiction treatment services where they felt judged, surveilled and stigmatized due to their drug use. This finding is consistent with other research on harm reduction programs for people who use drugs [50–53]. However, while participants highlighted the high quality of care they were receiving, many also highlighted that the medications being prescribed were insufficient. This is an area where some nuance is needed, as many participants were very satisfied with the medications they were receiving and the positive impacts this was having on their lives, including receiving medication doses that were preventing withdrawal and allowing for stability they had not previously attained from OAT. However, others highlighted difficulties in finding the right dose and/or right drug combinations to meet their needs; these difficulties are informative for future program development and improvement.

First, our analysis identifies two major and inter-related issues with the current pharmaceutical options available: the need for substitution with a wider range of substances that are stronger than the medications currently available, and the need for direct substitution with the actual substance people were using from street sources. While RMG in BC provided an enabling context for SAFER program implementation, it outlined limited options medications and dosing, and for individualized tailoring of prescriptions [20]. The SAFER program responded by developing their own clinical protocols, including beginning to prescribe fentanyl patches in 2021, which multiple participants in this study highlighted as being effective in meeting pain control needs. The ability of prescribers to use a wide variety of medication options and individualize dosing to meet individual tolerance and needs is common in other areas of medicine and ability to access a variety of doses and formulations of safer supply medications is likely crucial to effective safer supply prescribing. It will be equally crucial for programs to continue to adapt their medication offerings to align with the composition of substances in unregulated markets, to allow for effective dosing for participants. Many of the medications used in both OAT and safer supply programs are available on provincial formularies, and while universal pharmaceutical insurance coverage is not yet available across Canada, most provinces (including BC) have programs to ensure access to medications for people who could not otherwise afford to pay for them (i.e. people receiving social assistance or disability benefits). However, safer supply programs in many areas of Canada have faced difficulties in providing new formulations or medication options as many high dose opioids have been removed from formularies or are not covered by provincial pharmaceutical insurance plans [13, 54–56], leaving safer supply programs continuously behind the curve in attempting to meet participant needs. There is a need to ensure that the high dose opioids used within safer supply programs are available on provincial formularies and covered for all who need them.

Second, the lack of smokeable medication formulations was identified by participants as a continuing shortfall in safer supply programs. There is strong need for “right drug, right dose, right formulation” where a range of medication formulations are available to properly substitute substances from the unregulated supply including injectable, smokeable and oral options. In particular, there is a crucial need for smokeable fentanyl options to reflect how smoking fentanyl from unregulated markets has not just become a common method of administration (due to the potent high obtained), but also the most common method of administration identified during post-mortem investigations of drug toxicity fatalities in many jurisdictions [57]. In BC, smoking was identified as the mode of consumption in 71% of drug toxicity deaths in September 2023 [1]. This highlights the urgency of expanding availability smokeable options within safer supply programs.

Third, there is a continuing need for long term, sustained funding for prescribed safer supply programs. While safer supply prescribing grew following the beginning of the COVID-19 pandemic and subsequent roll-out of pilot funding for prescribed safer supply programs [3, 58], this federal pilot funding is temporary, and currently set to expire in 2025. It also reaches a very small number of people who are potentially at risk from the toxic drug supply; published data shows that among 70,360 people with opioid use disorder in BC, 5256 received an opioid prescription during the roll-out of risk mitigation prescribing (a form of safer supply prescribing) from March 2020 to August 2021 [24]. While there have been attempts to highlight the challenges associated with the temporary pilot funding [59], the permanent funding from provincial health budgets that is needed to maintain and expand prescribed safer supply has not been forthcoming, possibly due to continued contestation around safer supply in Canada [60].

A main strength of our study is the inclusion of people who use drugs as full partners in the research, allowing for their perspectives to be included in the entire research process. Limitations result from the restricted geographical setting of the research and pandemic-related restrictions that complicated data collection. Data were collected in a single urban centre with a highly toxic and volatile drug supply, and findings may not extend to other jurisdictions or rural/remote areas with different unregulated drug supplies and varying access to programs and services. Our sample consisted overwhelmingly of men who were unhoused, largely recruited and interviewed in encampments. The distribution of sample characteristics did not permit an examination of whether and how experiences varied by gender identity and ethno-cultural background, including Indigenous identity. Future research is needed to evaluate how intersecting identities affect access and experiences in safer supply programs. Finally, as all participants in this study were accessing safer supply, the perspectives of those who had accessed the program but stopped their participation are not reflected in the data.

## Conclusion

As safer supply programs continue to evolve as a response to the ongoing drug toxicity crisis, decision-makers and service providers are encouraged to be attentive to creating an environment that fosters partnership—with participants actively involved in navigating their care and able to express their needs and goals. As in other services and addiction treatment, trusting therapeutic relationships are crucial to promoting engagement and, by extension, positive outcomes. Particular attention is needed to ensuring access to an array of medication options and formulations, and to protocols for individualized dosing. The lack of direct replacements for the unregulated drugs that people consumed (e.g., primarily fentanyl, crystal methamphetamine and cocaine) can be expected to continue to hamper the effectiveness of safer supply programs.

## References

[1] BC Coroner’s Service. Unregulated Drug Deaths in B.C. 2023. Available from: https://www2.gov.bc.ca/gov/content/life-events/death/coroners-service/statistical-reports

[2] BC Coroner’s Service Death Panel Review. BC Coroner’s Service Death Panel Review: An Urgent Response to a Continuing Crisis. 2023 Nov 1; Available from: https://www2.gov.bc.ca/assets/gov/birth-adoption-death-marriage-and-divorce/deaths/coroners-service/death-review-panel/an_urgent_response_to_a_continuing_crisis_report.pdf

[3] Glegg S, McCrae K, Kolla G, Touesnard N, Turnbull J, Brothers TD (2022). “COVID just kind of opened a can of whoop-ass”: the rapid growth of safer supply prescribing during the pandemic documented through an environmental scan of addiction and harm reduction services in Canada. Int J Drug Policy.

[4] Gomes T, Kolla G, McCormack D, Sereda A, Kitchen S, Antoniou T (2022). Clinical outcomes and health care costs among people entering a safer opioid supply program in Ontario. CMAJ.

[5] Karamouzian M, Rafat B, Kolla G, Urbanoski K, Atkinson K, Bardwell G (2023). Challenges of implementing safer supply programs in Canada during the COVID-19 pandemic: a qualitative analysis. Int J Drug Policy.

[6] BC Centre on Substance Use. Heroin Compassion Clubs [Internet]. 2019. Available from: https://www.bccsu.ca/wp-content/uploads/2019/02/Report-Heroin-Compassion-Clubs.pdf

[7] Canadian Association of People who Use Drugs. Safe Supply Concept Document. 2019.

[8] Medrano K. DULF Keeps Distributing Checked Drugs as Health Canada Flags Denial [Internet]. Filter. 2022. Available from: https://filtermag.org/dulf-safe-supply-compassion-club-exemption/

[9] Wyton M. The Tyee. 2022 [cited 2023 Dec 21]. Health Canada Denies Compassion Club Exemption for Safe Supply. Available from: https://thetyee.ca/News/2022/09/01/Health-Canada-Denies-Compassion-Club-Exemption-Safe-Supply/

[10] Kalicum J, Nyx E, Kennedy MC, Kerr T (2024). The impact of an unsanctioned compassion club on non-fatal overdose. Int J Drug Policy.

[11] Drug User Liberation Front (DULF). 2023. DULF Compassion Club Preliminary Findings. Available from: https://www.dulf.ca/cc-preliminary-findings

[12] Macevicius C, Gudiño Pérez D, Norton A, Kolla G, Beck-McGreevy P, Selfridge M, et al. Just have this come from their prescription pad: the medicalization of safer supply from the perspectives of health planners in BC, Canada. Drugs: Education, Prevention and Policy. 2023;0(0):1–11.

[13] Kolla G, Long C, Perri M, Bowra A, Penn R. Safer Opioid Supply Program: Preliminary Report [Internet]. London: London Intercommunity Health Center; 2021 Nov p. 44. Available from: https://lihc.on.ca/wp-content/uploads/2022/01/2021-SOS-Evaluation-Full.pdf

[14] BC Centre on Substance Use. Risk Mitigation In the Context of Dual Public Health Emergencies. 2022. Available from: https://www.bccsu.ca/wp-content/uploads/2022/02/Risk-Mitigation-Guidance-Update-February-2022.pdf

[15] Atkinson K. Parkdale Queen West Community Health Center Safer Opioid Supply: 2023 Evaluation Report [Internet]. 2023 Mar. Available from: https://pqwchc.org/ programs-services/harm-reduction/safer-opioidsupply-sos-program

[16] Haines M, Tefoglou A, O’Byrne P. Safer Supply Ottawa Evaluation [Internet]. Ottawa; 2022 p. 55. Available from: https://safersupplyottawa.com/wp-content/uploads/SS-Ottawa-Evaluation-Report-Fall-2022.pdf

[17] Ivsins A, Boyd J, Mayer S, Collins A, Sutherland C, Kerr T (2021). “It’s helped me a lot, just like to stay alive”: a qualitative analysis of outcomes of a novel hydromorphone tablet distribution program in vancouver. Canada J Urban Health.

[18] McMurchy D, Palmer RW. Assessment of the Implementation of Safer Supply Pilot Projects [Internet]. Ottawa, Ontario; 2022 Mar p. 88. Available from: https://www.canada.ca/en/health-canada/services/opioids/responding-canada-opioid-crisis/safer-supply/early-findings-safer-supply-pilot-projects.html

[19] McNeil R, Fleming T, Mayer S, Barker A, Mansoor M, Betsos A (2022). Implementation of safe supply alternatives during intersecting COVID-19 and overdose health emergencies in British Columbia, Canada, 2021. Am J Public Health.

[20] Ranger C, Hobbs H, Cameron F, Stuart H, McCall J, Sullivan G, et al. Co/Lab Practice Brief: Implementing the Victoria SAFER Initiative [Internet]. Canadian Institute for Substance Use Research, University of Victoria; 2021 p. 12. Available from: https://www.uvic.ca/research/centres/cisur/assets/docs/colab/practice-brief-safer.pdf

[21] Schmidt RA, Kaminski N, Kryszajtys DT, Rudzinski K, Perri M, Guta A, et al. ‘I don’t chase drugs as much anymore, and I’m not dead’: Client reported outcomes associated with safer opioid supply programs in Ontario, Canada. Drug and Alcohol Review [Internet]. 2023 [cited 2023 Sep 25];n/a(n/a). Available from: https://onlinelibrary.wiley.com/doi/abs/10.1111/dar.13745 10.1111/dar.13745 37718646

[22] Slaunwhite A, Min JE, Palis H, Urbanoski K, Pauly B, Barker B (2024). Effect of Risk Mitigation Guidance opioid and stimulant dispensations on mortality and acute care visits during dual public health emergencies: retrospective cohort study. BMJ.

[23] Pauly B, McCall J, Cameron F, Stuart H, Hobbs H, Sullivan G (2022). A concept mapping study of service user design of safer supply as an alternative to the illicit drug market. Int J Drug Policy.

[24] Minkler M (2005). Community-based research partnerships: challenges and opportunities. J Urban Health.

[25] Minkler M (2010). Linking science and policy through community-based participatory research to study and address health disparities. Am J Public Health.

[26] Beck McGreevy P, Wood S, Thomson E, Burmeister C, Spence H, Pelletier J (2023). Doing community-based research during dual public health emergencies (COVID and overdose). Harm Reduct J.

[27] Harris M, Luongo N (2021). “Nothing about us, without us”: negotiating the personal and professional as activists and academics who use drugs. Int J Drug Policy.

[28] Nosyk B, Slaunwhite A, Urbanoski K, Hongdilokkul N, Palis H, Lock K (2021). Evaluation of risk mitigation measures for people with substance use disorders to address the dual public health crises of COVID-19 and overdose in British Columbia: a mixed-method study protocol. BMJ Open.

[29] Brothers TD, Leaman M, Bonn M, Lewer D, Atkinson J, Fraser J (2022). Evaluation of an emergency safe supply drugs and managed alcohol program in COVID-19 isolation hotel shelters for people experiencing homelessness. Drug Alcohol Depend.

[30] Lew B, Bodkin C, Lennox R, O’Shea T, Wiwcharuk G, Turner S (2022). The impact of an integrated safer use space and safer supply program on non-fatal overdose among emergency shelter residents during a COVID-19 outbreak: a case study. Harm Reduct J.

[31] BC Coroner’s Service. BC Coroners Service Death Review Panel: A Review of Illicit Drug Toxicity Deaths. 2022; Available from: https://www2.gov.bc.ca/assets/gov/birth-adoption-death-marriage-and-divorce/deaths/coroners-service/death-review-panel/review_of_illicit_drug_toxicity_deaths_2022.pdf

[32] Gomes T, Murray R, Kolla G, Leece P, Bansal S, Besharah J, et al. Changing Circumstances Surrounding Opioid-Related Deaths in Ontario during the COVID-19 Pandemic. ODPRN. 2021. Available from: https://odprn.ca/research/publications/opioid-related-deaths-in-ontario-during-covid/

[33] Frank D, Mateu-Gelabert P, Perlman DC, Walters SM, Curran L, Guarino H (2021). “It’s like ‘liquid handcuffs”: the effects of take-home dosing policies on methadone maintenance treatment (MMT) patients’ lives. Harm Reduct J.

[34] Simon C, Vincent L, Coulter A, Salazar Z, Voyles N, Roberts L (2022). The methadone manifesto: treatment experiences and policy recommendations from methadone patient activists. Am J Public Health.

[35] Walters SM, Perlman DC, Guarino H, Mateu-Gelabert P, Frank D (2022). Lessons from the first wave of COVID-19 for improved medications for opioid use disorder (MOUD) treatment: benefits of easier access, extended take homes, and new delivery modalities. Subst Use Misuse.

[36] Ivsins A, Boyd J, Mayer S, Collins A, Sutherland C, Kerr T (2020). Barriers and facilitators to a novel low-barrier hydromorphone distribution program in Vancouver, Canada: a qualitative study. Drug Alcohol Depend.

[37] Magel T, Matzinger E, Blawatt S, Harrison S, MacDonald S, Amara S, et al. How injectable opioid agonist treatment (iOAT) care could be improved? service providers and stakeholders’ perspectives. Drugs: Education, Prevention and Policy. 2023;1–12.

[38] Mayer S, Boyd J, Fairbairn N, Chapman J, Brohman I, Jenkins E (2023). Women’s experiences in injectable opioid agonist treatment programs in Vancouver, Canada. Int J Drug Policy.

[39] Riley F, Harris M, Poulter HL, Moore HJ, Ahmed D, Towl G (2023). ‘This is hardcore’: a qualitative study exploring service users’ experiences of Heroin-Assisted Treatment (HAT) in Middlesbrough, England. Harm Reduct J.

[40] Bonn M, Palayew A, Bartlett S, Brothers TD, Touesnard N, Tyndall M (2020). Addressing the syndemic of HIV, Hepatitis C, overdose, and COVID-19 among people who use drugs: the potential roles for decriminalization and safe supply. J Stud Alcohol Drugs.

[41] Marchand K, Beaumont S, Westfall J, MacDonald S, Harrison S, Marsh DC (2019). Conceptualizing patient-centered care for substance use disorder treatment: findings from a systematic scoping review. Subst Abuse Treat Prevent Policy.

[42] Figgatt MC, Salazar Z, Day E, Vincent L, Dasgupta N (2021). Take-home dosing experiences among persons receiving methadone maintenance treatment during COVID-19. J Subst Abuse Treat.

[43] Krawczyk N, Fawole A, Yang J, Tofighi B (2021). Early innovations in opioid use disorder treatment and harm reduction during the COVID-19 pandemic: a scoping review. Addict Sci Clin Pract.

[44] Krawczyk N, Rivera BD, Levin E, Dooling BCE (2023). Synthesising evidence of the effects of COVID-19 regulatory changes on methadone treatment for opioid use disorder: implications for policy. The Lancet Public Health.

[45] Meyer M, Strasser J, Köck P, Walter M, Vogel M, Dürsteler KM (2022). Experiences with take-home dosing in heroin-assisted treatment in Switzerland during the COVID-19 pandemic-Is an update of legal restrictions warranted?. Int J Drug Policy.

[46] Oviedo-Joekes E, Dobischok S, Carvajal J, MacDonald S, McDermid C, Klakowicz P (2023). Clients’ experiences on North America’s first take-home injectable opioid agonist treatment (iOAT) program: a qualitative study. BMC Health Serv Res.

[47] Gomes T, Campbell TJ, Kitchen SA, Garg R, Bozinoff N, Men S (2022). Association between increased dispensing of opioid agonist therapy take-home doses and opioid overdose and treatment interruption and discontinuation. JAMA.

[48] Min JE, Guerra-Alejos BC, Yan R, Palis H, Barker B, Urbanoski K (2024). Opioid coprescription through risk mitigation guidance and opioid agonist treatment receipt. JAMA Netw Open.

[49] Socias ME, Choi JC, Fairbairn N, Johnson C, Wilson D, Debeck K (2023). Impacts of the COVID-19 pandemic on enrollment in medications for opioid use disorder (MOUD) in Vancouver, Canada: an interrupted time series analysis. Int J Drug Policy.

[50] Kolla G, Tarannum CN, Fajber K, Worku F, Norris K, Long C (2024). Substance use care innovations during COVID-19: barriers and facilitators to the provision of safer supply at a toronto COVID-19 isolation and recovery site. Harm Reduct J.

[51] Pauly B. Chapter 9. Close to the Street: Nursing Practice with People Marginalized by Homelessness and Substance Use. In: Guirguis-Younger M, Hwang SW, McNeil R, editors. Homelessness & Health in Canada. Ottawa: Les Presses de l’Université d’Ottawa | University of Ottawa Press; 2017. p. 211–32. (Santé et société | Health and Society). Available from: http://books.openedition.org/uop/805

[52] Pauly B, Wallace B, Pagan F, Phillips J, Wilson M, Hobbs H (2020). Impact of overdose prevention sites during a public health emergency in Victoria, Canada. PLoS One.

[53] Pauly B (2008). Shifting moral values to enhance access to health care: Harm reduction as a context for ethical nursing practice. Int J Drug Policy..

[54] Kamal A, Ferguson M, Xavier JC, Liu L, Graham B, Lock K (2023). Smoking identified as preferred mode of opioid safe supply use; investigating correlates of smoking preference through a 2021 cross-sectional study in British Columbia. Subst Abuse Treat Prevent Policy.

[55] Kolla G, Fajber K. Safer Opioid Supply Program: A Comparison of SOS Client Outcomes From 2022 and 2023. Ontario, Canada: London Intercommunity Health Center; 2023. Available from: https://www.nss-aps.ca/sites/default/files/resources/2023-LIHC-SOS-Evaluation.pdf

[56] Xavier J, McGreevy P, McDougall J, Lamb J, A S, Haywood B, et al. Substance Use Patterns and Safer Supply Preferences Among People Who Use Drugs in British Columbia. 2023.

[57] MacDonald M, Cheng C, Wang T, McCormack D, Kolla G, Cahill TM (2023). Trends in varying modes of drug use in opioid toxicity deaths in Ontario from 2017 to 2021. Int J Drug Policy.

[58] Young S, Gomes T, Kolla G, McCormack D, Dodd Z, Raboud J (2023). Initiations of safer supply hydromorphone increased during the COVID-19 pandemic in Ontario: An interrupted time series analysis. PLoS One.

[59] Letter: More than 130 experts in substance use call on federal government to continue to support and scale-up safer supply programs. 2023. Available from: https://www.hivlegalnetwork.ca/site/letter-more-than-130-experts-in-substance-use-call-on-federal-government-to-continue-to-support-and-scale-up-safer-supply-programs/?lang=en

[60] Michaud L, Kolla G, Rudzinski K, Guta A (2024). Mapping a moral panic: news media narratives and medical expertise in public debates on safer supply, diversion, and youth drug use in Canada. Int J Drug Policy.

